# First molecular characterization of an Iranian population of *Schistonchus caprifici* (Gasparrini, 1864) Cobb, 1927 (Rhabditida; Aphelenchoidea)

**DOI:** 10.21307/jofnem-2020-039

**Published:** 2020-04-13

**Authors:** Hadi Karimipour Fard, Hamid Zare

**Affiliations:** 1 Plant Protection Research Department, Kohgiluyeh and Boyerahmad Agricultural and Natural Resources Research and Education Center, AREEO, Yasouj, Iran; 2 Fig Research Station, Fars Agricultural and Natural Resources Research and Training Center, AREEO, Estahban, Iran

**Keywords:** Aphelenchoidea, Fars province, phylogeny, taxonomy

## Abstract

A population of *Schistonchus caprifici* was recovered in Iran during the present study. The molecular characters of this population were studied using partial sequences of large subunit ribosomal DNA (LSU rDNA D2-D3) for the first time, and its phylogenetic relationships with other isolates of the species were discussed. Compared to previous available data, no remarkable genetic distance was observed between the presently studied isolate and other isolates.

Several representatives of Aphelenchoidea (Fuchs, 1937) have been reported from Iran, and currently the genera *Aphelenchoides* (Fischer, 1894), *Aprutides* (Scognamiglio et al., 1970), *Basilaphelenchus* (Pedram et al., 2018), *Cryptaphelenchus* (Fuchs, 1937), *Devibursaphelenchus* (Kakulia, 1967), *Ektaphelenchus* (Fuchs, 1937), *Ektaphelenchoides* (Baujard, 1984), *Laimaphelenchus* (Fuchs, 1937), *Robustodorus* (Andrássy, 2007), *Seinura* (Fuchs, 1931), and *Sheraphelenchus* (Nickle, 1970) are known that occur in the country (e.g. Pourjam and Bert, 2006; Pedram et al., 2011, 2012, 2018a, b; Atighi et al., 2012a, b; Moosavi et al., 2014; Miraeiz et al., 2015; Adeldoost et al., 2016; Pedram, 2017, 2019; Aliramaji et al., 2018, 2019, 2020; Mobasseri et al., 2018; Mirzaie Fouladvand et al., 2019a, b). *Schistonchus* Cobb, 1927 occurs in the country with only one representative, the type species, *Schistonchus caprifici* (Gasparrini, 1864) Cobb, 1927, being reported in three conference abstracts (Kheiri and Goldansaz, 1998; Pakniyat and Taghizadeh, 2000; Namjoo et al., 2019) with no morphological and morphometric data. However, the morphological characters and morphometric data were provided for an Iranian population of the species by Kolaei et al. (2016) from Kerman. The genera *Aprutides* and *Schistonchus* were not characterized from Iran with molecular data, unlike the other previously mentioned genera. Based on a recovered population of *S. caprifici* from syconia of *Ficus carica* collected from a fig garden in Estahban (Fars province) during the present study, the morphological and morphometric characters of this population were found to be in accordance with the data presented by Hunt (1993), Vovlas and Larizza (1996) and Kolaei et al. (2016). Therefore, the present study aimed to characterize this recently recovered population for its molecular identity by sequencing the D2-D3 expansion fragments of the large subunit of the ribosomal DNA gene, as the first molecular analysis of the Iranian population of the species. The two LSU fragments from two female specimens were amplified and sequenced to yield identically aligned 705 and 717 nt long sequences (GenBank accessions MN634962 and MN634963, respectively). Their BLAST search revealed they have 99.15 to 100% identity with the D2-D3 sequences of other isolates of the species deposited into GenBank. To reconstruct the LSU tree, several previously sequenced populations and the sequences generated by Gulcu et al. (2008) were selected. The two most divergent sequences from Turkey in the latter study (EU287674 and EU287689) were also included. Within the selected sequences, the newly generated sequences had 100% identity with the sequences of the populations from Turkey, Spain, and Italy (e.g. EU287643-EU287665-FN564936-EU287646). Aligning of the two newly generated sequences with two divergent sequences in the study of Gulcu et al. (2008) revealed they have six indels compared to EU287674 and eight indels compared to EU287689. In the previous study by Gulcu et al. (2008), a 0 to 13 different nucleotides range was observed between different populations of the species from Turkey. The genetic distance between selected D2-D3 sequences of *S. caprifici* calculated using MEGA6 (Tamura et al., 2013) ranged from 0.001 (between EU287679 and EU287653) to 4.90 (between EU287692 and GU190765).

In the Bayesian phylogenetic tree inference under the GTR + I + G evolutionary model (Fig. 1), the newly generated sequences fell into the maximally supported clade of *S. caprifici*. This is the first molecular phylogenetic study of the species from Iran, showing D2-D3 sequences of the studied Iranian population is identical to those of the majority of previously sequenced populations.

**Figure 1:**
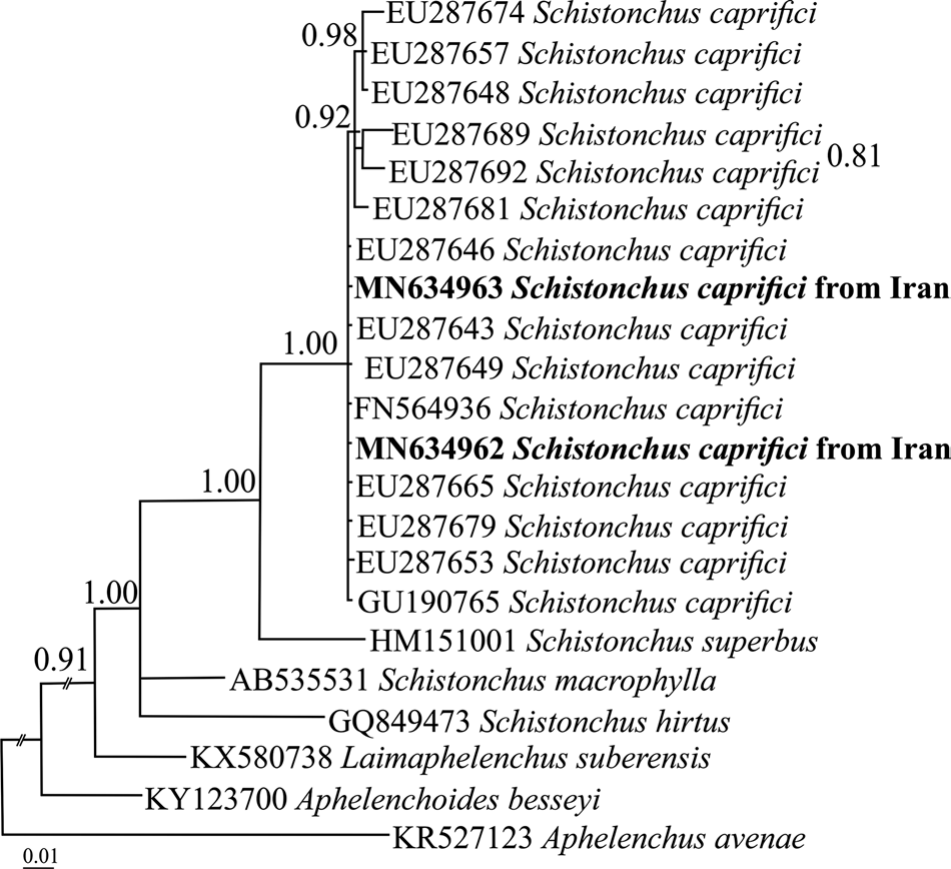
Bayesian 50% majority rule consensus tree inferred from D2-D3 large subunit (LSU) rDNA gene sequences of Iranian population of *Schistonchus caprifici* (Gasparrini, 1864) Cobb, 1927 under the GTR + I + G model. The newly generated sequences are in bold font. The accession numbers in clade of *S. caprifici* starting with “EU” are from Turkey, the two other accession numbers starting with “FN” and “GU” are from Italy or Spain.
